# Layered Signaling Regulatory Networks Analysis of Gene Expression Involved in Malignant Tumorigenesis of Non-Resolving Ulcerative Colitis via Integration of Cross-Study Microarray Profiles

**DOI:** 10.1371/journal.pone.0067142

**Published:** 2013-06-25

**Authors:** Shengjun Fan, Zhenyu Pan, Qiang Geng, Xin Li, Yefan Wang, Yu An, Yan Xu, Lu Tie, Yan Pan, Xuejun Li

**Affiliations:** 1 State Key Laboratory of Natural and Biomimetic Drugs, Department of Pharmacology, School of Basic Medical Sciences, Peking University and Beijing Key Laboratory of Tumor Systems Biology, Peking University, Beijing, China; 2 Department of Pharmacy, Xi’an Children’s Hospital, Xi’an, China; 3 Department of Cardiology, Peking University People’s Hospital, Beijing, China; 4 Pediatric Research Institute, Qilu Children’s Hospital of Shandong University, Jinan, China; Queen’s University Belfast, United Kingdom

## Abstract

**Background:**

Ulcerative colitis (UC) was the most frequently diagnosed inflammatory bowel disease (IBD) and closely linked to colorectal carcinogenesis. By far, the underlying mechanisms associated with the disease are still unclear. With the increasing accumulation of microarray gene expression profiles, it is profitable to gain a systematic perspective based on gene regulatory networks to better elucidate the roles of genes associated with disorders. However, a major challenge for microarray data analysis is the integration of multiple-studies generated by different groups.

**Methodology/Principal Findings:**

In this study, firstly, we modeled a signaling regulatory network associated with colorectal cancer (CRC) initiation via integration of cross-study microarray expression data sets using Empirical Bayes (EB) algorithm. Secondly, a manually curated human cancer signaling map was established via comprehensive retrieval of the publicly available repositories. Finally, the co-differently-expressed genes were manually curated to portray the layered signaling regulatory networks.

**Results:**

Overall, the remodeled signaling regulatory networks were separated into four major layers including extracellular, membrane, cytoplasm and nucleus, which led to the identification of five core biological processes and four signaling pathways associated with colorectal carcinogenesis. As a result, our biological interpretation highlighted the importance of EGF/EGFR signaling pathway, EPO signaling pathway, T cell signal transduction and members of the BCR signaling pathway, which were responsible for the malignant transition of CRC from the benign UC to the aggressive one.

**Conclusions:**

The present study illustrated a standardized normalization approach for cross-study microarray expression data sets. Our model for signaling networks construction was based on the experimentally-supported interaction and microarray co-expression modeling. Pathway-based signaling regulatory networks analysis sketched a directive insight into colorectal carcinogenesis, which was of significant importance to monitor disease progression and improve therapeutic interventions.

## Introduction

As the fourth commonest carcinoma, colorectal cancer (CRC) associated with significant cause of mortality worldwide owing to its prevailing distant metastasis [1], [2]. Unfortunately, there are still more than 783,000 new cases diagnosed and roughly 394,000 deaths yearly [3]. Further, it is conservatively estimated the lethality will continue to rise for the increased life-expectancy and aging population [4], [5]. Epidemiological studies uncovered individuals consistently exposed to inadequate physical practices or high-fat dietary closely interrelated with high risk of colorectal neoplasia [6]. Besides, environmental and heritable factors also made significant contributions to CRC susceptibility [7]. Traditional pathological examination have identified several causative modifiers, including *TP53*, *K-ras, APC, Wnt5, beta-catenin, DCC* or microRNAs [8], [9]. Among them, *APC* and *DCC* were the most frequently detectable prognostic signatures for CRC; however, the results were perplexed and cardinal hurdles for clinical therapeutic interventions were still insurmountable. Since most of variant genes have ineffectual profits for diagnosis and underlying mechanisms associated with CRC are ill-defined.

Considerable documents certified countless malignancies occurred in association with chronic inflammation. Infection with hepatitis B or C viruses had been found to be the main cause of hepatocellular carcinoma [10], [11]. Besides, inflammation also directly related to DNA methylation and epithelial cell malignant transformation [12], [13]. As an inflammatory response to infection, inflammation-mediated CRC had been reviewed [14]. Numerable evidences pointed out chronic ulcerative colitis (UC) was intimately connected with colorectal carcinogenesis [15], [16], [17], [18]. However, our current knowledge regarding signaling regulatory networks between UC and CRC has not been unraveled yet.

Cells in multi-cellular organisms switch into diverse fates, such as division, proliferation, apoptosis or differentiation into specialized phenotypes. Genome-wide association studies (GWAS) of gene regulatory networks (GRNs) govern this process and the inference of GRNs is crucial for understanding underlying molecular mechanisms between genes and gene regulation [19], [20]. Typically, GRNs are modeled as a structure of genes, *cis*-elements, and regulators. The regulators, also called transcription factors, are often described as proteins which bound to specific regions of target gene, and thereby regulated the transcription of genetic information from DNA to mRNA [21]. They functioned as an activator or repressor alone or with other proteins to regulate the recruitment of RNA polymerase to targets [22], [23]. Moreover, apart from proteins, microRNAs also participate in the transcriptional and post-transcriptional regulatory manner of gene expression in plants and animals [24]. In addition, interactions among regulators are defined as *cis*-elements in GRNs and thereby control the level of gene expression during transcription.

As yet, a wide variety of approaches have been proposed for modeling GRNs, such as discrete models of Boolean networks [25], [26], Bayesian networks [27], [28], system of equations [29], continuous models of neural networks [30], association network [31], and co-expression models [20], etc. Among them, the most successful approaches for GRNs inference were ascribed to the reconstruction of the association network from target predictions [32]. However, the so-called ‘guilt-by-association’ methods [33], [34] need long time consumption, making them unsuitable for GRNs inference in term of large-scale genomes [35]. Meanwhile, owing to its high risk of false-positive noise, cardinal drawback for the association network modeling was its weak correlations, which was insufficient to elaborate the real connections within the network. Needless to say, with the rapid increase of experimental approaches, the advent of microarray facilitated large-scale monitoring of gene expression under different conditions at one time and enlightened roles of genes associated with infirmities in a systematic visualization [36], [37]. Thus, inference of gene regulations in GRNs based on microarray gene co-expression models provided an opportunity for understanding the underlying regulatory mechanism [38]. Meanwhile, knowledge associated with subcellular localization in a network is indispensable for grasping molecular function and intricate biological pathway at subcellular level [39]. Thus, modeling of layered GRNs by integrating large-scale microarray expression data sets contributed to elucidate illnesses in a systems biology perspective.

However, mining these data to better comprehend gene expression and regulation proposes a major challenge for bioinformatics. Since practical considerations restrain the size of samples and the overlap genes in multiple studies are limited with poor predictability [40]. If small sizes of individual studies from different experiments are combined to increase sample size, the integrative manner is therefore a promising approach and a more accurate reconstruction of GRNs can be expected [41]. Nevertheless, real biological variation from the data set still exists [42] when different samples are added to an existing one or in a meta-analysis of multiple studies that pools microarray data across different laboratories or platforms [43]. To make up the scarcity, we perform a simulation by randomly generated 8 virtual data sets, also called random microarray data sets (RDSs), from the integrative microarray cohort.

The purpose of functional genomics in the post-genome era is to better elucidate molecular mechanisms involved in gene regulation [44]. Genes assigned to certain gene sets by clustering analysis belong to different regulatory modules or signaling pathways. GRNs clarify the interaction among transcription factors and inference of transcriptional regulatory networks assists in understanding underlying mechanism of complex cellular processes or responses [45]. Given that gene-gene interactions contribute to complex diseases, the combination of multiple variants based on biological pathways tends to uncover the synergistic effects of large-scale genes and highlight the specific signaling pathways involved in diseases [46].

Traditionally, cell signaling is described as linear diagrams. As more cross-talk between signaling has been reviewed, a systematic network view of cell signaling is proposed [47]. Among them, one of the pioneering attempts to model signaling network was assigned to the map of human cancer signaling. In Cui *et al.* network, a comprehensive analysis of human cancer signaling architectural organization assembled from cancer-associated genetically and epigenetically altered genes was established [48]. In addition, Fan and colleagues also performed a network-based pathway analysis using gene co-expression models to specify the off-target effects for torcetrapib. They highlighted that IL-2 Receptor Beta Chain in T cell Activation, Platelet-Derived Growth Factor Receptor (PDGFR) beta signaling pathway, IL2-mediated signaling events, ErbB signaling pathway and signaling events mediated by Hepatocyte Growth Factor Receptor (HGFR, c-Met) were answered for the adverse cardiovascular effects associated with torcetrapib [49]. Thus, pathway-based signaling regulatory networks had been widely applied to complex diseases, which led to identifying diseases-susceptibility pathways for therapeutic interventions [50], [51], [52].

In this study, we modeled a layered signaling regulatory network associated with colorectal cancer from cross-study microarray gene expression data using the experimentally-supported interaction and microarray co-expression modeling. Cytoscape [53] in association with four plugins including BisoGenet [54], NetworkAnalyzer, AllegroMCODE and Cerebral [55] was applied for inferring the layered signaling regulatory network. To our knowledge, by far, there were no published documents expounded the pathological transition focusing on a layered signaling network and our study provided infrequent insights into the potential molecular mechanisms, which might be useful for colorectal cancer therapeutic prevention or intervention.

## Materials and Methods

### Microarray Data Sets Selection

We search the public functional genomics data repositories including ArrayExpress (http://www.ebi.ac.uk/arrayexpress/), Gene Expression Omnibus (GEO, http://www.ncbi.nlm.nih.gov/geo/) and Stanford Microarray Database (SMD, http://smd.stanford.edu/) for microarray data sets satisfying the following criteria: (1) deriving from Homo sapiens; (2) depicting genome-wide co-expression information of ulcerative colitis and neoplastic lesions; (3) supplying raw data files. After extensively retrieval, two different microarray expression cohorts performed on Affymetrix GeneChip platform were downloaded from GEO and selected for further investigation (Table 1).

**Table 1:** Microarray data sets utilized in this experiment.

Authors	Documents (PMID)	GEO number	Array type	Number of samples
**Gyorffy ** ***et al.***	20087348	GSE4183	Affymetrix Human Genome U133 Plus 2.0 Array	30(IBD:15; CRC:15)
**Pekow ** ***et al.***	23388545	GSE37283	Affymetrix HT HG-U133+ PM Array	15(UC:4;CRC:11)

Abbreviations: IBD: inflammatory bowel disease, CRC: colorectal carcinoma, UC: ulcerative colitis.

The first data set obtained from Gyorffy *et al*. (GEO ID: GSE4183) was performed on Affymetrix Human Genome U133 Plus 2.0 Array platform [56]. This study derived from different stages of pathophysiological background of colonic diseases with 15 samples for inflammatory bowel disease (IBD) and 15 samples for colorectal carcinoma (CRC) with the purpose of developing a comprehensive comparison of preprocessing algorithms on samples to afford a data warehouse which can be further mined for in-depth pathway analyses.

The other one is a subset of 15 Affymetrix HT HG-U133 GeneChips originated from a gene expression experiment by Pekow *et al.* [57]. The aim of this original investigation was to perform a genome-wide expression profiling between chronic ulcerative colitis (UC, 4 arrays) and neoplastic lesions (11 arrays) to ultimately detect underlying prognostic gene signatures involved in this pathophysiological transition (GEO accession number GSE37283). All clinical specimens utilized in this study were directly taken from patient-sufferers either surgically disconnected or during surveillance colonoscopy.

### Computational Pipeline

To standardize the microarray data sets obtained from independent studies and reduce systematic distortions produced by different laboratories, rank normalization was introduced to effectuate robust multi-array average (RMA) analysis [58]. Subsequently, platform comparison from Affymetrix probe identifier to NCBI AILUN [59] was performed and cross-study normalization was achieved using ArrayMining (http://www.arraymining.net/), which pooled multiple microarray data from independent investigations or platforms into a combinational cohort [60].

### Simulated Gene Expression Data Analysis

We simulated 8 virtual cohorts, called the random microarray data sets (RDSs), from the integrated data set mentioned above [42]. For each RDSs, we created at least 12 samples. Afterwards, all the RDSs were performed Significance Analysis of Microarray (SAM, http://www-stat.stanford.edu/~tibs/SAM/) to produce a cluster of up- or down-regulated variant genes via comparison of UC with CRC [61]. Gene expression was regarded as significantly dissimilar if the threshold of false discovery rate (FDR) less than 0.05 and fold change above 1.2.

### Cancer Signaling Regulatory Map Construction

To provide a high-quality human cancer signaling atlas with great superiority, the manually curated molecules of human cancer signaling were assembled as previously described [48]. Briefly, a map of human cancer signaling was firstly obtained from Cui *et al.*, which united manually curated signaling molecules including BioCarta (http://www.biocarta.com/) [62], literature-mined signaling network [63], Cancer Cell Map including cancer mutated genes obtained from COSMIC database and other high-throughput profiling, methylated genes in cancer stem cells, cancer associated gene set acquired from plasmID (http://plasmid.hms.harvard.edu/) and Online Mendelian Inheritance in Man (OMIM, http://www.ncbi.nlm.nih.gov/omim). Subsequently, Human Protein Reference Database (HPRD, http://www.hprd.org/) [49] and REACTOME (http://www.reactome.org/) databases were appended. After adding connections between signaling molecules based on SysBiomics platform (http://biomine.cigb. edu.cu/sysbiomics/) [54], the original human cancer signaling map was visualized by Cytoscape (http://www.cytoscape.org/) [53]. Dispersive nodes without interactions and small sub-networks were discarded afterwards. Only the largest component was considered as the map of human cancer signaling. Meanwhile, duplicated edges and interaction direction were abandoned from the network utilizing NetworkAnalyzer plugin before analysis. Ultimately, the reconstructed human cancer signaling network was clustered by AllegroMCODE using Molecular Complex Detection (MCODE) clustering algorithm [64].

### Layered Signaling Regulatory Network Analysis

Subcellular localization information involved in the signaling regulatory networks driven by the co-differently-expressed genes was diffusely retrieved from Human Protein Reference Database (HPRD, http://www.hprd.org/), Human Proteinpedia (http://www.humanproteinpedia.org/) and EntrezGene (http://www.ncbi.nlm.nih.gov/gene/). Cerebral generated a view of the layered map afterwards and each of the signaling regulatory networks was divided into four layers, including extracellular, membrane, cytoplasm and nucleus [55]. Nodes without localization information were positioned into the same layer of their adjacent neighbors.

### Gene Ontology (GO) and Pathway Analysis

Functional enrichment analysis was performed to evaluate underlying biological functions involved in the layered signaling regulatory networks. In this study, the DAVID functional annotation clustering tool (http://david.abcc.ncifcrf.gov/) was freely employed to enrich the potential biological functions based on “GOTERM_BP_FAT” option [65], [66]. For pathway enrichment analysis, ToppCluster (http://toppcluster.cchmc.org/) was selected as gene sets category [67].

### Hierarchical Signaling Regulatory Networks Organization

To obtain an intuitionistic two-dimensional graphical representation of large-scale cluster members across an activity profile and define the main clusters by visual inspection of the resulting tree, R (http://www.r-project.org/) cooperated with stats package was introduced to hierarchically trace heatmap for the over-represented GO biological processes and signaling pathways in the layered signaling regulatory network.

## Results

Two independent microarray data sets depicting UC and CRC from clinical specimens were utilized in this experiment. RMA analysis was applied to implement rank normalization based on WebArray [58]. Platform comparison from Affymetrix probe identifier to NCBI was performed using AILUN [59] before cross-study normalization and integration [60]. Totally, there were 20,083 common genes, 455 unique genes in GPL570, and 433 diverse genes in GPL13158. We then performed a simulation by randomly generating 8 RDSs from the combinational cohort and carried out SAM to produce a cluster of variances.

To acquire high-quality human cancer signaling, we manually curated cancer signaling molecules thoroughly [48], [49]. All the over-represented genes obtained from the virtual RDSs were mapped to portray the context-specific sub-communities and the remodeled signaling regulatory networks were redistributed according to their subcellular localization. To better expound the underlying molecular mechanisms associated with CRC initiation in the layered signaling networks, the DAVID functional annotation tool and ToppCluster were freely employed to implement Gene Ontology (GO) and pathway enrichment analysis, respectively. In general, the remodeled signaling regulatory networks mainly responded to negative regulation of DNA recombination, nucleotide-excision repair, modification-dependent protein catabolic process, protein catabolic process and modification-dependent macromolecule catabolic process. Pathway enrichment analysis indicated that EGF/EGFR signaling pathway, EPO signaling pathway, T cell signal transduction and members of the BCR signaling pathway were primarily responsible for the malignant transition of CRC.

### Identification of Co-differently-represented Genes Associated with Colorectal Cancer

**Figure 1:**
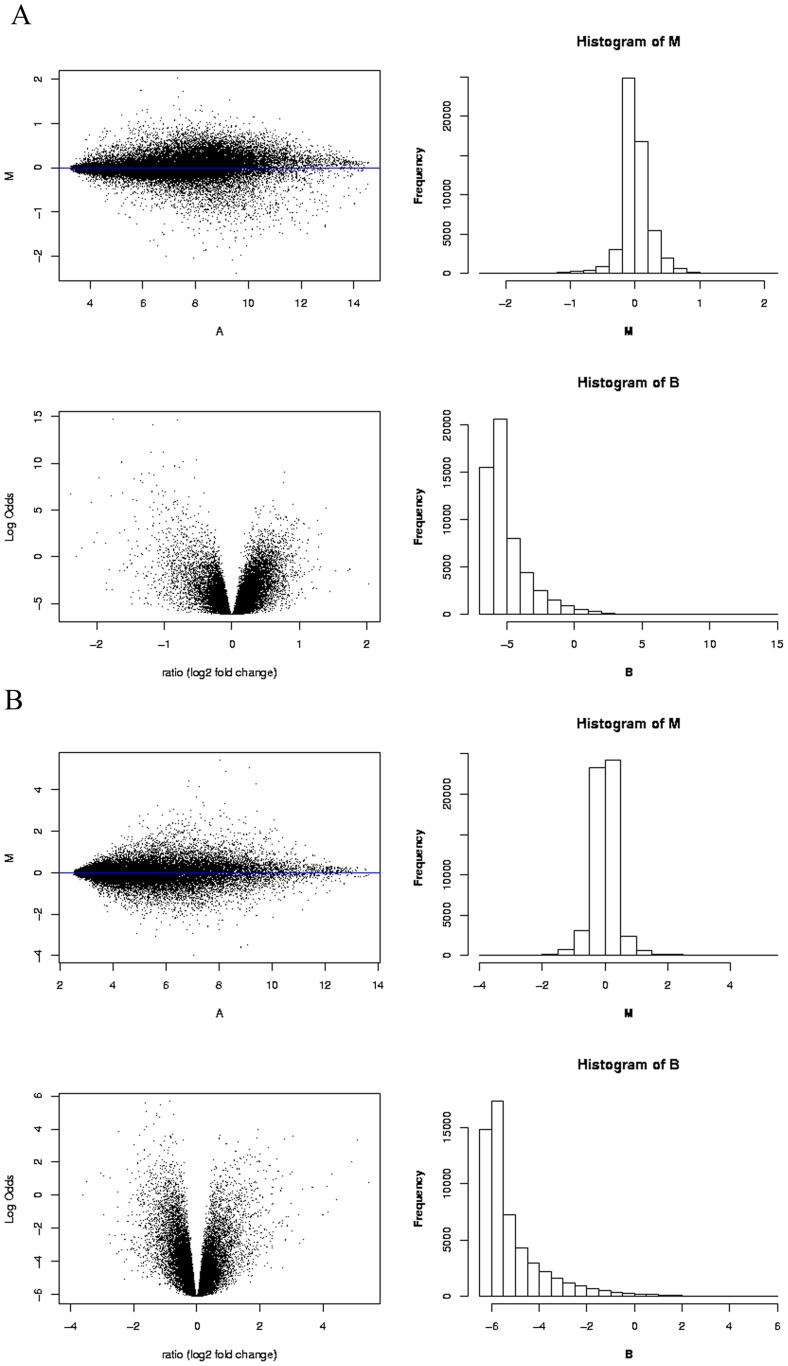
Robust multi-array average (RMA) analysis results of microarray data based on WebArray. (A) Statistical analysis result plot for GSE4183 included M-A plot, M-B plot, M histogram and B statistics histogram. (B) Statistical analysis result plot for GSE37283 included M-A plot, M-B plot, M histogram and B statistics histogram. M: the log-differential expression ratio; A: the log-intensity of spot, a measure of overall brightness of spot; B: B statistics, the log-odds of differential expression.

**Figure 2:**
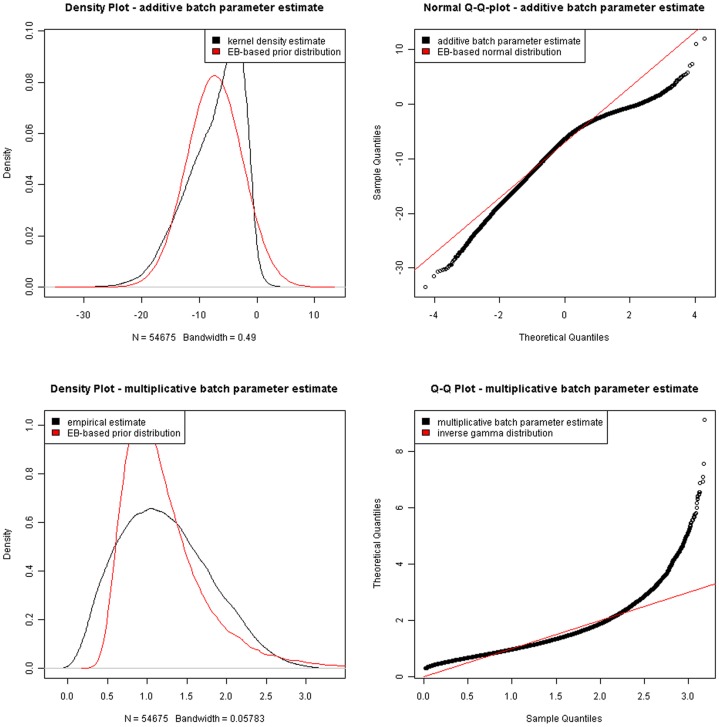
Cross-study normalization and integration results of two separate microarrays based upon ArrayMining.

To identify variant regulators between UC and CRC, two separate microarray data sets with primary intensity data files of Affymetrix CEL format were submitted to WebArray to carry out RMA analysis (Appendix S1 and S2). The output graphic plots including histogram, M-A plot and M-B plot for each study before and after within-array normalization were presented in Figure 1. We then effectuated cross-study normalization and integration using ArrayMining, which averaged across individual probe expression values and the integrative data set were attached in Appendix S3 and S4. Figure 2 pictured the density plot and Q-Q plot before and after integration based on Empirical Bayes (EB) algorithm, which focused on pooling independent microarray data sets utilizing parametric and non-parametric EB approach to filter out batch effects [68]. Finally, 8 RDSs originated from the same combinational cohort were performed SAM to produce a list of differently expressed genes for false discovery rate (FDR)<0.05 and the fold change>1.2 (Appendix S5).

### Human Cancer Signaling Network Construction

To infer a map of human cancer signaling, varieties of repositories were exhaustively retrieved. Functional relations between nodes were added using BisoGenet in SysBiomics. In total, the reconstructed human cancer signaling network consisted of 11,728 nodes and 94,471 connections, and the raw data was appended in Appendix S6.

### Signaling Regulatory Networks Organization

**Figure 3:**
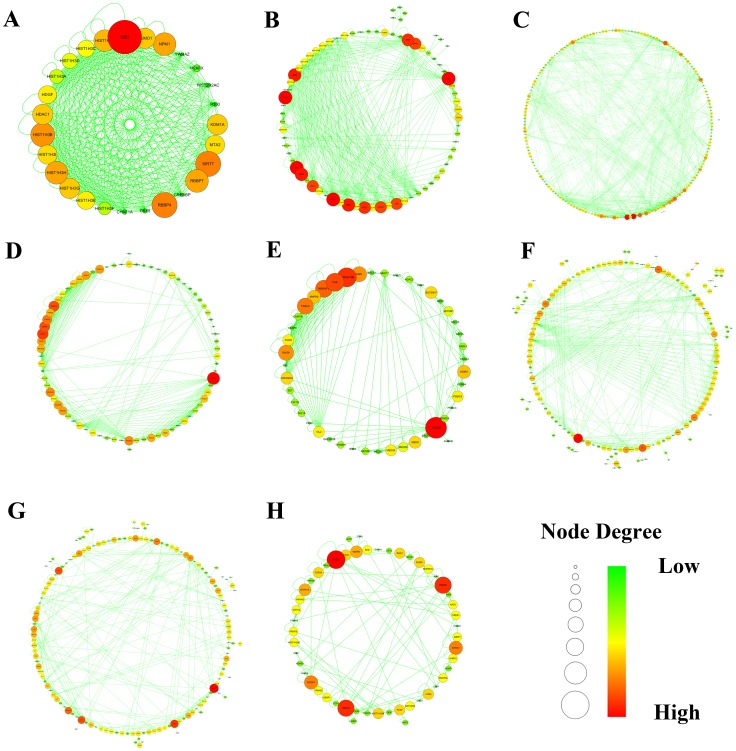
Signaling regulatory modules of human cancer signaling network generated by AllegroMCODE based on molecular complex detection (MCODE) algorithm. A-H represented signaling regulatory networks 1–8. Circle dots in the networks corresponded to genes. Red represented high degree connectivity, whereas green stood for low degree connectivity.

We next sought to pursue the decisive signaling regulatory cohort associated with the aggressive transition from UC to CRC in the map of human cancer signaling using MCODE clustering algorithm [64]. As presented in Figure 3, 8 network modules with a cluster score above 2.0 were detected. Subsequently, all the differently expressed regulators were mapped to portray the context-specific signaling sub-communities. As shown in Table 2, out of the 8 signaling regulatory modules, 3 gene sets originating from cluster 3, 6 and 7 were principally driven by most of the variance and answered for colorectal carcinoma initiation.

**Table 2:** Simulated results for the random microarray data sets (RDSs).

Rank of RDSs	Virtual microarray data sets	Drivergenes	Signalingnetworks
1	IBD(GSM95520,95513,95522,95518,95521,95517); CRC(GSM915466,915461,95499,95510,915465,95501)	CDK6,CEBPD,SOCS1,CD19,BCL6	3,6,7
2	IBD(GSM915451,915452,95512,95523,95515,95524,95514);CRC(GSM91546,95502,915463,915470,95506,95498)	PTGS2	6
3	IBD(GSM95515,95511,95517,95521,95513,95518); CRC(GSM95496,95497,915470,95508,95500,95509)	NEDD4L	3
4	IBD(GSM95520,95523,915452,95514,915454,95525); CRC(GSM915469,915465,95505,95506,95502,95510)	SUMO1,SYT1,SOX2,PTGS2	1,3,6,7
5	IBD(GSM95512,95520,915453,95519,95511,95524); CRC(GSM95509,95507,95510,915464,915468,915467)	STK4,MORF4L2	6,7
6	IBD(GSM95518,915452,95516,95525,95522,91515); CRC(GSM915469,95500,95496,915461,95499,95497)	STAT1	6
7	IBD(GSM915451,95523,95522,95515,915453,95521); CRC(GSM915469,915464,95504,95503,915467,915460)	PTGS2	6
8	IBD(GSM915451, 915452, 915453, 915454); CRC(GSM915460, 915461, 915462, 915463, 915464, 915465, 915466, 915467, 915468, 915469,915470)	CXCR4,SMAD3,PTGS2,PTPRC,BCL6,TLR4	3,6,7

Abbreviations: IBD: inflammatory bowel disease, CRC: colorectal carcinoma.

### Layered Signaling Regulatory Networks Construction

**Figure 4:**
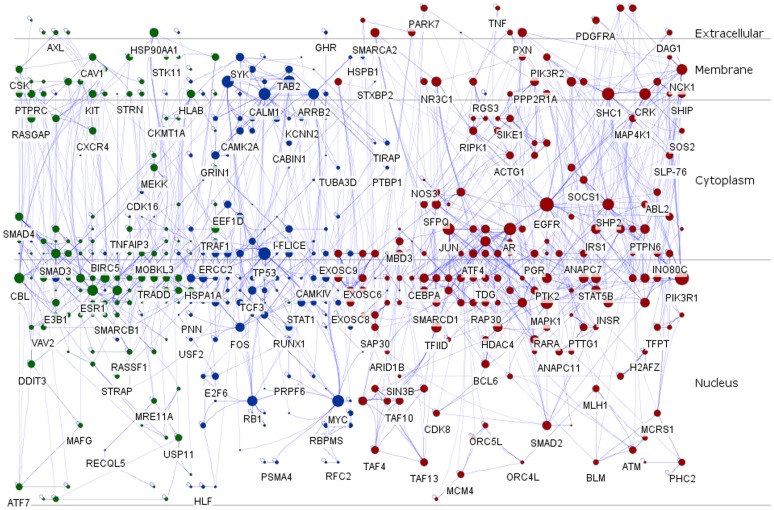
Layered signaling regulatory networks driven by co-differently-expressed microarray genes involved in malignant transition of colorectal cancer from the benign chronic non-solving ulcerative colitis to the more aggressive one. In the layered signaling regulatory networks, the size of each node was proportional to the degree. In addition, red, blue and green represented nodes stemmed from signaling networks 3, 6 and 7, respectively.

Subcellular localization information involved in the context-specific sub-communities encoded by the virtual RDSs was retrieved from HPRD, Human Proteinpedia or EntrezGene, and imported as node attributes. Afterwards, Cerebral automatically generated a view of the layered signaling regulatory network which integrated 3 signaling regulatory sub-networks according to the subcellular localization, and the remodeled signaling regulatory networks were divided into four layers, including extracellular, membrane, cytoplasm and nucleus (Figure 4). Besides, nodes in the layered network were proportionate to degree and empowered polychrome schemes to distinguish these modules. Red and blue represented nodes located in cluster 3 and 6, respectively. Whereas, green stood for hubs scattered in cluster 7.

### GO Analysis

**Figure 5:**
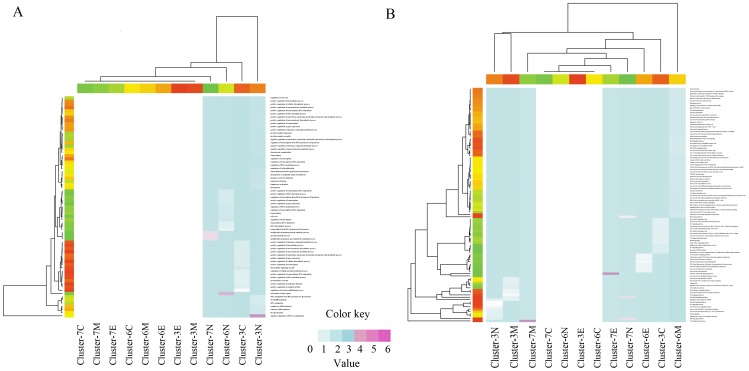
Heatmap of the over-represented biological processes and enrichment pathways using stats package in R environment. For each figure, columns correspond to biological processes (A) or signaling pathways (B), and rows correspond to gene cluster category and subcellular localization. Expression values are logarithm of ratio value utilizing log transform data. Red and blue in each grid represented positive, while white represented null. (A) Significantly over-represented GO biological processes in differential cluster and layers. (B) Significantly enriched signaling pathways in differential cluster and layers. E: Extracellular. M: Membrane. C: Cytoplasm. N: Nucleus.

The layered signaling network associated with CRC in the context of GO was assessed by DAVID to identify significantly over-represented biological functions (FDR<0.01). Meanwhile, a heatmap with hierarchically clustering was produced to visualize these processes based on stats package in R project. As shown in Figure 5 A, most of the biological processes related to molecular metabolic, modification, biosynthetic, transcription and catabolic processes. Particularly, we underlined the importance of regulators in nucleus layer, which indicated that negative regulation of DNA recombination, nucleotide-excision repair, modification-dependent protein catabolic process, protein catabolic process and modification-dependent macromolecule catabolic process were responsible for the pathological transition from UC to colorectal carcinogenesis.

### Pathway Analysis

To gain a detailed insight into the functions of the whole regulators in the layered signaling regulatory network, we additionally performed pathway enrichment analysis using ToppCluster. As appended in Figure 5 B, genes distributed in extracellular, membrane and nucleus regions of signaling network 7 were highly associated with CRC initiation especially for EGF/EGFR signaling pathway, EPO signaling pathway, T cell signal transduction and members of the BCR signaling pathway (FDR<0.01).

## Discussion

As microarray data on gene expression programs become available, it is profitable to create a systematic view of biological systems to improve our understanding of underlying mechanisms associated with disorders [69], [70]. Given that extensive investigations of signaling had been studied over the past few decades, knowledge concerning signaling regulation had been assembled and deposited in publicly available resources. Since the abnormal expression of genes involved in diseases frequently resulted in genome instability, analysis of gene-gene interactions in the context of gene regulatory networks could reveal the specific GRNs that led to the dysfunction of biological systems [71]. Thus, an integrative analysis of human cancer signaling network based on cross-study microarray expression profiles was of great availability in CRC initiation and progression [48].

Despite massive amounts of experimentally-supported microarray expression data bestowed in public repositories, it was still strenuous to avail legitimately. Since experiments were run on different laboratories, the combined usage of multiple platforms was necessary to overcome technical variation of individual study that resulted from sample preparation, labeling, hybridization, handling and other processing steps [72]. For the purpose of the study, a cross-study multiple microarray data integration and normalization method were applied to infer signaling pathways involved in colorectal carcinogenesis. Recently, Li *et al.* indicated the ‘one-step-clustering’ of gene expression profiles and network-based gene signatures used in the past decade was far from sufficient to produce robust gene signatures [42]. To overcome the deficiency and offer robust and accurate modulated signatures, we carried out a simulation by randomly generating a cohort of virtual microarray data set gained from the combinational one. Even though each of the RDSs was subsets of the identical cohort, the variance varied diversely. Of note, we discovered 3 signaling networks including cluster 3, 6 and 7 turned out to be robust, suggesting the stimulated re-sampling from different combinations of microarray cohort could effectively cut down the false-positive signatures in the noise.

In this study, we modeled a layered signaling regulatory network via integration cross-laboratories microarray expression data based on the experimentally-supported interaction and microarray co-expression modeling. Pathway-based signaling regulatory networks analysis revealed EGF/EGFR signaling pathway, EPO signaling pathway, T cell signal transduction and members of the BCR signaling pathway were involved in colorectal carcinogenesis. Our biological interpretations are as follows:

### EGF/EGFR Signaling Pathway

Numerous experimental and epidemiological investigations supplied strong evidences that EGF/EGFR signaling pathway played a pivotal role in ulcer repair. As a single-chain polypeptide of 53 amino acid residues, epidermal growth factor (EGF) could stimulate cell growth, proliferation and differentiation through autocrine, paracrine and endocrine mechanisms. It had been indicated that EGF could accelerate ulcer repair in the experimental colitis animal model [73], resulting in the alteration of downstream signaling cascades and subsequently catalyzing preferential substrates mediated by DAG, IP3 and phosphorylated RAS [74]. Recent studies demonstrated EGF could promote goblet cell mucus secretion and protect rat jejunal mucosa from injury induced by mechanical trauma [75]. Meanwhile, Luck *et al*. also discovered that EGF could significantly reduce colon ulcer and inflammation *in vivo* after intracavitary application, suggesting EGF was a protective cytokine in ulcerative colitis [76].

However, the over-expression of EGF was an aggressive biological behavior, as increased levels of EGF/EGFR were detected in innumerable types of carcinomas [77], [78]. In other words, high levels of EGF or the activation of EGF/EGFR pathway had been found to significantly associate with tumor initiation and proliferation. Numerous signaling cascades including *KRAS/BRAF* and *PI3K/AKT* were certified to entangle in colorectal cancer [79]. In the presence of EGFR, the accumulation of EGF in solid tumor led to the activation of the *PI3K/AKT* pathway which subsequently gave rise to solid tumor proliferation. Therefore, tactics targeting EGF/EGFR cascade system would have been an effective and timely approach for clinical prevention and therapeutic intervention for the transition from chronic non-resolving UC to pernicious colorectal carcinoma [80].

### EPO Signaling Pathway

Pathway-based approach for analysis of GWAS also proposed the assumption that the dysfunction of EPO signaling pathway was assigned to be a highly aggressive biological process associated with colorectal carcinogenesis. Erythropoietin (EPO) was a primary growth factor regulating erythroid progenitor proliferation and maturation [81]. Despite insufficient evidences concluded, several recent investigations still clung to provide infrequent clues that EPO signaling pathway was closely linked to IBDs. Pro-inflammatory cytokines, such as interleukin-1beta (IL-1beta), tumor necrosis factor alpha (TNF-alpha), and interferon gamma (INF-gamma), are produced in increased amounts by peripheral-blood monocytes and mononuclear cells in intestinal lamina propria in patients with IBD [82], [83]. *In vitro* and *in vivo* models indicating administration erythropoietin could drastically reverse the suppression related to pro-inflammatory cytokines of cellular maturation of the erythroid lineage [84], [85]. What’s more, Lee *et al.* discovered recombinant human erythropoietin (rhEPO) treatment remarkably attenuated hyperoxia-induced lung injury by down-regulating inflammatory responses in neonatal rat model [86]. It has been indicated that hypoxia and necrosis were common features of ulcerative colitis [87], [88]. The elevated hypoxia-inducible factors (HIFs) level induced by hypoxic conditions in ulcerative colitis was a direct catalyst which accelerated the synthesis and release of EPO [89]. According to the documented literatures, erythropoietin deficiency contributed to the development of chronic anemia in patients with IBD [90]. Further, in patients combined with refractory anemia and IBD, treatment with oral iron and rhEPO could raise hemoglobin and improve hematocrit [91]. Thus, high levels of EPO in ulcerative colitis led to the activation of EPO signaling pathway and therefore served as an anti-inflammatory factor against inflammation progression.

The roles of EPO had been well documented in erythrocytopoiesis, but its clinical relevance in carcinoma remained controversial and deserved for further investigations. Activation of EPO signal transduction pathway was significantly dysregulated during disorders progression to a more aggressive phenotype. As early as 2003, EPO was suggested as a rewarding guideline for cancer patients. However, owing to an observed higher mortality, concerns were raised that EPO was unprofitable for survival in cancer patients [92]. In 2003, Henke and his colleagues pointed out EPO was prosperous to correct anemia in head-and-neck cancer patients; however, it failed to improve, and even impair, cancer patient-sufferers [93]. In addition, high levels of EPO and EPO receptor (EPOR) were found in various cancer cell types and the EPO/EPOR system was known to induce proliferation, angiogenesis and even inhibit apoptosis [94]. Yasuda *et al.* examined the expression of the EPO/EPOR in 24 kinds of malignant cancerous cell lines and confirmed EPO signal transduction system was engaged in tumorigenesis of almost all malignancies [95]. Meanwhile, Mohyeldin and Lai also attested EPO was a crucial clinical signatures for head and neck squamous cell carcinoma diagnosis [96], [97]. Analogous conclusions could also be obtained in lung cancer [98], prostate cancer [99] and ovarian cancer [100], which indicated EPO/EPOR signaling system was tightly connected with tumor cell apoptosis, hypoxia resistance and metastasis. Furthermore, it had been confirmed that cancerous cell lines with EPO pretreatment rendered them less sensitive to the cytotoxicity of cisplatin [95]. In general, EPO signaling pathway appeared to be considerably altered in the malignant transition from ulcerative colitis to colorectal carcinogenesis.

### T Cell Signal Transduction

Imbalance of anti-inflammatory mediators (e.g., IL-10 and TGF) and excessive pro-inflammatory responses appeared to be a risk factor for chronic IBD [101]. Mice with IL-10-KO or lymphocyte-deficient Rag-KO were reported to develop spontaneous IBD [102]. On the contrary, several of the pro-inflammatory factors had been identified to associate with T cell production, such as IL-12, IL-23, IL-6, as well as IL-17. Of note, elevated production of memory CD4^+^ regulatory T cells (Treg) specifically stimulated by IL-23 is especially relevant to tissue inflammation [102], [103]. In the case of IBD models mentioned above, memory CD4^+^ Treg constituted 60–80% of the whole T cells in mice with UC [104]. In addition, Fiona *et al*. also discovered that CD4/CD45RB^high^ Treg could be confirmed as an initiator of IBD, suggesting T cell signal transduction was highly associated with chronic IBD [105].

Several studies had shown a huge accumulation of Treg distributed in solid tumors and increased with the transition from benign to a malignant state [106]. Large numbers of cells with the phenotype of Treg in patients with early stage lung cancer have implications in the pathogenesis [107]. A recent study in mice revealed that the efficacy of therapeutic cancer vaccination in mice could be enhanced by removing CD4^+^CD25^+^ Treg, suggesting T cell signal transduction played a role in immunosuppressive effect. Meanwhile, activated suppression by Treg had been confirmed to play an important role in T cells down-regulation [106]. Thus, interventions including selective depletion Treg, inhibition of the proliferation and immune regulation of Treg or suppression Treg from tumor microenvironment aggregation, might be an available choice [108], [109]. In brief, T cell signal transduction was found to play an essential role during the transition from a benign UC to a malignant CRC.

### Members of the BCR Signaling Pathway

B cell receptor (BCR), a multi-protein complex with an antigen binding subunit and a signaling subunit, was crucial for inflammatory signaling initiation and propagation. With the assistance of costimulatory signals such as natural killer (NK) or M cytokines, B cell superantigen (BSAg) could interact with B cells and Ig, and participate in immune system or inflammatory disorders via cross-linking with BCR [110], [111]. BSAg could enlarge immune systems via activation of autoreactive B cells which subsequently led to the amplification of local or systemic inflammatory reaction through interacting with BCR [112]. Silverman *et al*. uncovered, owing to the damage involved in intestinal digestive tract, polyclonal activators could mediate local immune response through penetrating lymphoid tissues [113].

Aberrant BCR signaling pathway plays significant roles in the pathogenesis of tumor immunity. Recent studies demonstrated that over-expression of regulatory B cell (Breg) could enhance tumor immunity [114]. As a member of the FOX protein family involved in immune system response, FOXP3 (forkhead box P3) was a master regulator in the development of Treg [115]. Elevated levels of Breg disturbed in tumor could transform CD4^+^ Treg into FoxP3^+^Treg via direct interaction with transforming growth factor-beta (TGF-beta) pathways, resulting in tumor proliferation. However, in the absence of Breg, the transformation of FoxP3^+^Treg was impeded and inhibited breast cancer metastasis [116]. Thus, in view of the negative regulation of Breg, depletion of B cells might be propitious to enhance the anti-tumor capacity [117] and the application of rituximab could significantly reduce the number of mature B cells in body, which resulted in an inhibitory effect on colorectal carcinoma initiation and progression [118].

### Conclusions

A layered signaling regulatory network involved in the transition from primary UC to aggressive CRC was successfully modeled via linking cross-study microarray co-expression models with experimentally-supported interactions from the map of human cancer signaling, which led to identifying several chief cellular processes and signaling pathways. These pathological processes had been documented to be characteristics for colorectal carcinogenesis, and could be summarized into four main pathways, including EGF/EGFR signaling pathway, EPO signaling pathway, T cell signal transduction and members of the BCR signaling pathway. Therefore, our biological interpretation was focused on these pathways and their potential contributions to the transition from benign UC to aggressive or malignant CRC.

Our model for inferring the layered signaling regulatory network has two key points. For one thing, the connections between nodes or hubs in the signaling regulatory networks were integrated from cross-laboratories microarray expression profiling. For another, the layered signaling regulatory network, as opposed to the traditional ones, provided infrequent insights into molecular function and the intricate signaling pathways at the subcellular level. However, due to its positive connections and some noises that could not be avoided, the layered signaling regulatory network presented here is still not comprehensive. Nevertheless, we still confirmed that our inference would provide incentive illustration for the malignant transition of CRC, and supplied directive significance for future therapeutic intervention.

## Supporting Information

Appendix S1 **Robust multi-array average (RMA) analysis result of GSE37283 based upon WebArray platform.**
(ZIP)

Appendix S2 **Robust multi-array average (RMA) analysis result of GSE4183 based upon WebArray platform.**
(ZIP)

Appendix S3 **Cross-study normalization and integration results (part 1) using ArrayMining.**
(ZIP)

Appendix S4 **Cross-study normalization and integration results (part 2) using ArrayMining.**
(ZIP)

Appendix S5 **Detailed list of the differently expressed genes by Significant Analysis of Microarray (SAM) originated from the simulated microarray data sets or virtual random microarray data sets (RDSs).**
(ZIP)

Appendix S6 **A map of human cancer signaling network.**
(ZIP)
